# Gender and sex—time to bridge the gap

**DOI:** 10.15252/emmm.201910668

**Published:** 2019-04-12

**Authors:** Gian‐Paolo Dotto

**Affiliations:** ^1^ Department of Biochemistry University of Lausanne Epalinges Switzerland

**Keywords:** Genetics, Gene Therapy & Genetic Disease

## Abstract

Interdisciplinary research and education are key for a better appreciation of sex and gender as defining a person, and for novel societal and personalized health‐care approaches.

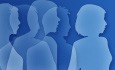

Interdisciplinary research and education are key for a better appreciation of sex and gender as defining a person, and for novel societal and personalized health‐care approaches. While the involvement of abnormal Androgen Receptor (AR) signalling in prostate cancer is well established, there is growing evidence that it also plays a role in various other malignancies. A key target organ for epigenetic modification is the human brain, which has anatomical differences in various regions between men and women. While significant research is underway to better understand the factors that determine sex and gender, other essential health‐related issues for transgender patients are much less well studied.

Sex and gender are different characteristics and yet inextricably linked to the human experience in determining bodily, mental and social activities. Sex refers to the biological and physical features linked with reproductive functions that result from the combined action of sex chromosomes and hormones. Gender refers more widely to an individual's self‐perception and representation as male, female or non‐binary and their place in society. Historically, the health sciences have kept sex and gender separated as biological and societal factors. However, there are increasing biological as well as medical arguments to question whether there is a true divide between the two. Personalized prevention and treatment of a large number of medical conditions critically depends on a better appreciation of both the physiological difference between the biological sexes and how male, female and non‐binary identities are attained and maintained. In fact, sex and gender are at the interface between environmental and hormonal factors and genetic and epigenetic determinants of organ development, most notably the brain. Interdisciplinary research and education are key for a better appreciation of sex and gender as defining a person, and for novel societal and personalized health‐care approaches.

## The role of the Y

It is commonly known that the presence of the male Y chromosome triggers a cascade of events resulting in male sex development, with female determination occurring by default (Wijchers & Festenstein, 2011; Arnold, 2012). Less appreciated is the fact that the genetic information carried by the Y chromosome is minuscule relative to that stored in the X chromosome and that only the single *SRY* gene, buried in a condensed dormant region of the Y chromosome, drives the whole male sex determination process (Waters *et al*, 2007). The SRY‐coded protein, also called the testis‐determining factor (TDF), is expressed for only a brief moment during embryo development. Its function is to bend the curvature of the DNA at a specific site on a separate chromosome, leading to the persistently elevated expression of a second protein, SOX9. This transcription factor is responsible for converting initially equivalent cells, present in what will become the kidney secretory apparatus (*mesonephros*), into cells of the male gonads. SOX9 is also expressed during development in many other parts of the embryo, in both males and females, and fulfils a number of functions unrelated to sex determination.

There are many clinical examples of the importance of this sex‐determining axis. Patients with loss of either the *SRY* or *SOX9* gene have a fully female phenotype even if they carry the Y chromosome (Waters *et al*, 2007; Wijchers & Festenstein, 2011; Arnold, 2012). Conversely, patients with translocation of the *SRY* gene to an autosomal chromosome exhibit a male phenotype in spite of carrying two X chromosomes. It can be argued that these are rare genetic syndromes that can be largely ignored by the general population, even within the medical profession. However, clinical syndromes with similar dissociation between sex chromosomes and male versus female phenotypes occur as a result of inactivating mutations of another gene of key importance for many other aspects of human health and behaviour: the gene coding for the AR (Hughes *et al*, 2012). This protein, expressed in cells of all types and functions, senses and responds to androgens, steroid hormones named for their “male‐determining” function. Testosterone, and its more potent form dihydrotestosterone (DHT), is the quintessential androgen produced by specialized testicular cells, and it instructs the development of all secondary male sex organs and other male phenotypic characteristics, ranging from musculoskeletal mass to behavioural traits. Inactivating mutations of the AR gene, or of the gene encoding for the enzyme involved in testosterone modification (5α‐reductase), result in individuals with a non‐binary or female phenotype even in the presence of a XY genotype and testosterone‐producing testicles (Hughes *et al*, 2012).

## A dance of hormones

Importantly, AR fulfils pleiotropic functions besides sex determination and is activated by a variety of other androgens besides testosterone, which are produced in both sexes. Diminishing levels of androgens contribute significantly to age‐associated decline of multiple functions. Changes in AR activity also bridge the divide between the two sexes in disease. While the involvement of abnormal AR signalling in prostate cancer is well established, there is growing evidence that it also plays a role in various other malignancies—specifically breast and lung cancer (Clocchiatti *et al*, 2016).

The localization of the AR gene on the X chromosome also dispels any notion that AR fulfils only male‐determining functions. While random X chromosome inactivation ensures that AR expression is similar in both sexes, males with a single copy of the gene are twice more susceptible to deleterious mutations (Hughes *et al*, 2012). The Y chromosome is also not neutral, as a number of genes on it, including *SRY*, may function as positive determinants of AR expression and/or activity. In this context, AR signalling could be preferentially affected by the frequent and poorly understood loss of the Y chromosome that occurs in the male population, in ageing tissues and cancer cells (Clocchiatti *et al*, 2016).

As mentioned above, the absence of the SRY‐SOX9 regulatory axis will result in the formation of ovaries instead of testicles, while the production of female sex hormones, specifically estrogens, drives secondary female sex organ development. Estrogens play an opposite role to androgens in many other biological contexts and, like androgens, are secreted in both men and women. Perhaps less known is the biochemical and functional interconnection and, to some extent, interchangeability between male and female sex hormones. Estrogens are metabolically derived from androgens, while derivates of another key female hormone, progesterone, can exert androgenic functions.

The end point of this complex interplay between sex chromosomes and hormones is at the level of epigenetic modifications (Wijchers & Festenstein, 2011). Compared to the more permanent differences established during embryonic development, epigenetic marks are established later in life and can be partially reversed. A key target organ for epigenetic modification is the human brain, which has anatomical differences in various regions between men and women (Hines, 2011). These are not associated with any attitudinal and cognitive differences of statistical significance between the two sexes. By contrast, there seems to be a link with gender self‐representation, which can already be documented in children of very young age, using game preferences as a reliable parameter (Hines, 2011).

## Effects on brain development

From all of the above, it follows that “gender”, as a person's self‐representation as male or female, may or may not coincide with “sex”, as a more biological dimension characterized by sex chromosomes, gonads and resulting anatomical features. Differences in specific brain regions have been associated with gender self‐representation, including the third interstitial nucleus of the anterior hypothalamus, (INAH‐3), which tends to be larger in males than in females (Hines, 2011).

How these anatomical differences are connected with gender self‐representation and associated sex orientation remains a mystery. What seems clear, however, is the role of androgens as endogenous/exogenous factors that determine gender self‐representation as early as during embryonic development, with effects that may persist throughout the entire life (Hines, 2011; Goldstein *et al*, 2019). Adding an additional twist of complexity, androgens have been reported to exert their impact on brain development directly or indirectly, through their conversion into estrogens. Irrespective of the detailed mechanisms, the self‐representation of children born of mothers with endocrine syndromes has led to the conclusion—supported by parallel studies of experimental animal systems—that exposure of the developing embryo to elevated androgen levels leads to male self‐identification and female self‐identification in the opposite case.

The early impact of androgens on brain development can be modified or reinforced later in life by a variety of endogenous and exogenous factors, but it is hard to revert (Hines, 2011; Goldstein *et al*, 2019). An irreversible set of events occurring during embryonic life may be at the basis of gender dysphoria, a partial or complete dissociation between the gender self‐representation of an individual and his/her genetic and gonadal sex (Wanta & Unger, 2017). Those who identify as transgender may therefore choose to affirm their gender by hormonal or surgical intervention to alter their biological sex. However, it is important not only for gender identity clinics to assist with psychological and social transition, along with surgical and hormonal changes to physical characteristics.

## Addressing sex‐ and gender‐related health issues

While significant research is underway to better understand the factors that determine sex and gender, and how to alleviate gender dysphoria, other essential health‐related issues for transgender patients are much less well studied (Wanta & Unger, 2017). There are some data on the long‐term impact of cross‐sex hormones in transgender patients, though further exploration is required. Similarly, the consequences on age‐associated incidence and progression of various cancer types, immune/inflammatory diseases and cardiovascular function are severely under‐studied, in spite of the major impact that androgens and estrogens have in all these contexts. Also important is to understand the effects of androgens and other substances with sex hormone activity to which the developing human embryo is exposed. Endocrine disruptors, chemical substances that are ubiquitous in the environment and that have the ability to block, induce or interfere with the activity of endogenous sex hormones, can be easily transferred from the mother to the developing embryo with effects that persist into adult life (Messerlian *et al*, 2017).

Addressing these challenges will require more communication and collaboration among molecular biologists who study sex‐determining molecules and their effects on human physiology and behaviour with specialists who are familiar with gender‐related psychological and societal issues. Interdisciplinary research, a generally recognized and attractive principle, is key for such an endeavour, but there are scant support mechanisms. It can be fairly stated that molecular biologists and geneticists very rarely if ever read journals or attend meetings focused on psychological, social or environmental issues and *vice versa*. Epidemiology, classically defined as “the study of the distribution and determinants of health‐related states or events”, may hold the key to bridging this gap. I am a basic molecular/cell biologist, and it is from epidemiologists that I learnt of the substantially different susceptibility of men versus women to various cancer types, and the unmet need to investigate the long‐term health risks of prolonged exposure to exogenous and endogenous sex hormones and related compounds.

Moreover, as a teacher, I also feel that we have a specific responsibility to point out such challenges and questions and to raise awareness for the next generation of scientists. While it is essential for students and researchers to stay focused at an early stage of their career in order to be effective and move forward, scientists are increasingly called on to tackle societal issues. Young researchers’ drive and enthusiasm to explore the unknown is an invaluable treasure; they, even more than their senior colleagues, should be encouraged to leave the comfort zone of established research fields and venture into new territory. Disentangling the complex relationship between sex and gender, between genes and hormones, self‐perceptions, disease risk and social attitudes and expectations would be a worthy challenge to take on.
